# Exploring age and gender variations in root canal morphology of maxillary premolars in Saudi sub population: a cross-sectional CBCT study

**DOI:** 10.1186/s12903-024-04310-w

**Published:** 2024-05-09

**Authors:** Mohmed Isaqali Karobari, Azhar Iqbal, Rumesa Batul, Abdul Habeeb Adil, Jamaluddin Syed, Hmoud Ali Algarni, Meshal Aber Alonazi, Tahir Yusuf Noorani

**Affiliations:** 1 Department of Dental Research, Center for Global Health Research, Saveetha Medical College, and Hospitals, Saveetha Institute of Medical and Technical Sciences, Saveetha University, Chennai, Tamil Nadu India; 2 Department of Restorative Dentistry & Endodontics, Faculty of Dentistry, University of Puthisastra, Phnom Penh, 12211 Cambodia; 3 Department of Restorative Dentistry, College of Dentistry, Jouf University, Sakaka, 72345 Saudi Arabia; 4 Director Research & Development, OWA Medical and Research Center, Sugarland, TX USA; 5 Oral Basic and Clinical Sciences, Faculty of Dentistry, King Abdulaziz University, p.o box, Jeddah, 80209 Saudi Arabia; 6 Conservative Dentistry Unit, School of Dental Sciences, Universiti Sains Malaysia, Health Campus, Kubang Kerian, 16150 Kota Bharu, Kelantan Malaysia; 7 Saveetha Dental College, Saveetha Institute of Medical and Technical Sciences (SIMATS), Chennai, Tamil Nadu India

**Keywords:** Cone beam computed tomography, Dental anatomy, Dental diagnostic imaging, Dental pulp, Endodontics, Morphology, Root, Root canal, Premolars

## Abstract

**Background:**

In complex teeth like maxillary premolars, endodontic treatment success depends on a complete comprehension of root canal anatomy. The research on mandibular premolars’ root canal anatomy has been extensive and well-documented in existing literature. However, there appears to be a notable gap in available data concerning the root canal anatomy of maxillary premolars. This study aimed to explore the root canal morphology of maxillary premolars using cone-beam computed tomography (CBCT) imaging, considering age and gender variations.

**Methods:**

From 500 patient CBCT scans, 787 maxillary premolar teeth were evaluated. The sample was divided by gender and age (10–20, 21–30, 31–40, 41–50, 51–60, and 61 years and older). Ahmed et al. classification system was used to record root canal morphology.

**Results:**

The most frequent classifications for right maxillary 1st premolars were ^2^MPM^1^ B^1^ L^1^ (39.03%) and ^1^MPM^1^ (2.81%), while the most frequent classifications for right maxillary 2nd premolars were ^2^MPM^1^ B^1^ L^1^ (39.08%) and ^1^MPM^1^ (17.85%). Most of the premolars typically had two roots (left maxillary first premolars: 81.5%, left maxillary second premolars: 82.7%, right maxillary first premolars: 74.4%, right maxillary second premolars: 75.7%). Left and right maxillary 1st premolars for classes ^1^MPM^1^ and ^1^MPM^1–2−1^ showed significant gender differences. For classifications ^1^MPM^1^ and ^1^MPM^1–2−1^, age-related changes were seen in the left and right maxillary first premolars.

**Conclusion:**

This study provides novel insights into the root canal anatomy of maxillary premolars within the Saudi population, addressing a notable gap in the literature specific to this demographic. Through CBCT imaging and analysis of large sample sizes, the complex and diverse nature of root canal morphology in these teeth among Saudi individuals is elucidated. The findings underscore the importance of CBCT imaging in precise treatment planning and decision-making tailored to the Saudi population. Consideration of age and gender-related variations further enhances understanding and aids in personalized endodontic interventions within this demographic.

## Introduction

The morphology and variability of root canal systems play a crucial role in the success of endodontic treatment [1, 2]. Understanding the intricacies of root canal anatomy is essential for effective diagnosis, treatment planning, and applying appropriate techniques. The research on mandibular premolars’ root canal anatomy has been extensive and well-documented in existing literature [3, 4]. However, there appears to be a notable gap in available data concerning the root canal anatomy of maxillary premolars [5–9].

Maxillary premolars present unique challenges due to their anatomical complexity, including multiple canals, isthmuses, and accessory canals [10, 11]. Accurately identifying and classifying root canal systems in maxillary premolars is crucial for diagnosis and achieving optimal treatment outcomes [12].

Despite the importance of understanding root canal morphology, there remains a gap in knowledge concerning maxillary premolars. This lack of comprehensive information on the root canal morphology of maxillary premolars hinders endodontic practitioners’ ability to deliver precise and successful treatments [13]. This study aims to fill this gap by conducting an investigation using cone-beam computed tomography (CBCT) imaging. CBCT, as a non-invasive and highly accurate imaging technique, offers the advantage of providing detailed three-dimensional representations of root canal systems, which were previously not easily achievable through conventional radiographs [14]. The high-resolution images obtained through CBCT will provide valuable data to enhance the knowledge and clinical management of root canal anatomy in these teeth, leading to better-informed treatment decisions and reduced complications [4, 15].

By analyzing a large sample size of CBCT images, we aim to comprehensively understand the root canal configuration in maxillary premolars, considering factors such as age and gender [16]. The findings of this study will contribute to enhancing the knowledge and clinical management of root canal anatomy in maxillary premolars, improving treatment success rates, and reducing complications.

By elucidating the variations and complexities of root canal morphology in maxillary premolars, this study will aid dental professionals in making informed decisions regarding treatment approaches, instrument selection, and the application of advanced endodontic techniques [17, 18]. Furthermore, the results will provide valuable insights for dental educators, researchers, and students, facilitating the development of standardized protocols and guidelines for managing root canal systems in maxillary premolars.

## Methodology

### Study design

This study employed a retrospective cross-sectional design to comprehensively investigate the root canal morphology of maxillary premolars using cone-beam computed tomography (CBCT) imaging. This design allows for the examination of a large sample size and facilitates the analysis of root canal anatomy variations among different age groups and genders. By retrospectively analyzing CBCT images, the study aimed to elucidate the complex root canal anatomy of maxillary premolars and identify potential factors influencing their variability.

### Ethical consideration

Ethical approval was obtained from the Local Committee of Bioethics for Research at the Dentistry College, King Abdul-Aziz University (Ethical Approval No. 025-02-22). Informed consent was obtained from the Committee of Bioethics for Research, College of Dentistry, King Abdul-Aziz University, Jeddah, Saudi Arabia, considering the retrospective nature of the study. This ensured that the study adhered to ethical standards and protected the rights and confidentiality of the participants. Additionally, the study complied with all relevant regulations and guidelines regarding the use of patient data for research purposes.

### Sample size determination

The sample size for this study was determined using G Power 3.1.9.4 software, considering a chi-square test for goodness-of-fit, statistical power analysis, and an a priori approach. A comprehensive sample of 500 patient records was obtained, resulting in the evaluation of 787 maxillary premolar teeth. This large sample size enhances the statistical power of the study and allows for robust analysis of root canal morphology variations. It also increases the generalizability of the findings to the target population.

### Inclusion and exclusion criteria

Inclusion criteria were carefully defined to ensure the selection of appropriate teeth for analysis. Healthy maxillary premolars with small carious or restorative crowns, fully formed root apex, and defect-free radiographic images were included in the study. Exclusion criteria were applied to eliminate potential confounding factors, including root canal-treated teeth, fractured upper and lower posterior teeth, post and core restorations, calcification, resorption defects, and anomalies of crown and root. These criteria helped ensure the homogeneity of the study sample and the validity of the results.

### Imaging technique

CBCT images were acquired using the iCAT scanner system (Imaging Sciences International, Hatfield, PA, USA), a widely recognized and reliable imaging device in dentistry. Standardized imaging parameters (120 KVp, 5–7 mA) were employed to ensure consistent image quality across all scans. The use of CBCT allowed for the acquisition of detailed three-dimensional representations of root canal anatomy, enabling precise analysis and classification. High-resolution images obtained through CBCT provided valuable data for evaluating root canal morphology.

### Calibration and reliability

Prior to data collection, calibration was conducted involving an expert endodontist and an observer. The observer underwent rigorous training to accurately identify and classify root canal morphology. Calibration involved the examination of 50 CBCT images, with discrepancies resolved through discussion to achieve consensus. The kappa test was utilized to determine the level of agreement between observers, and intra- and interobserver reliability was assessed. Furthermore, specimens were assessed independently by observers following calibration to minimize bias and ensure consistency in the evaluations. A high kappa value (0.8) was obtained, indicating substantial to almost perfect reliability, thereby ensuring the validity of the data collected. This rigorous calibration process helped minimize observer bias and enhance the reliability of the study findings.

### Root and canal analysis

**Fig. 1:**
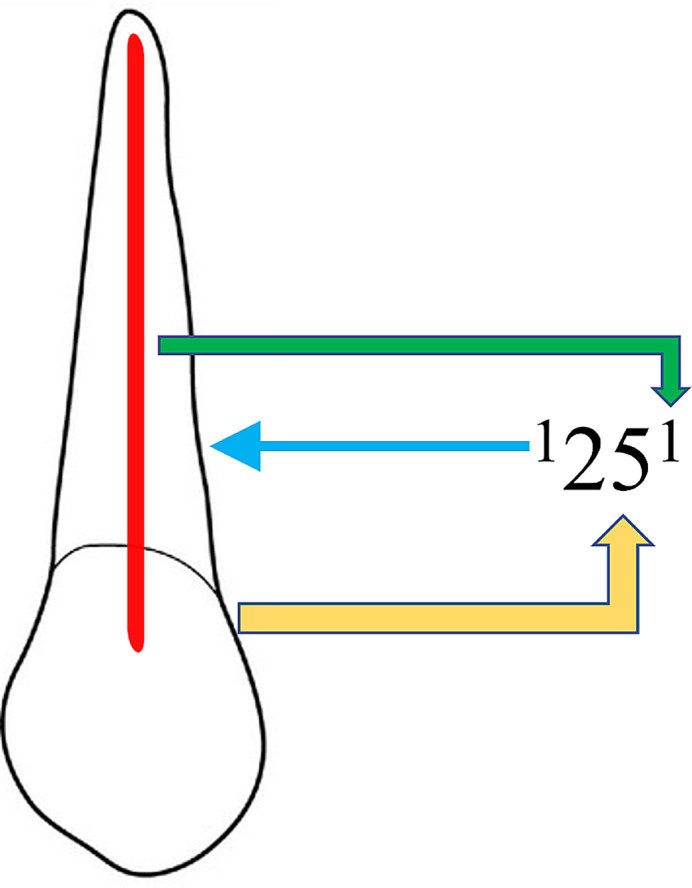
New classification system for root canal morphology of maxillary left second premolar classified using the new classification system, described as code ^1^25^1^. The code consists of three components, the tooth number – Yellow color arrow, number of roots – blue color arrow and the root canal configuration – green color arrow. The number of roots is added as a superscript before the tooth number, so it is single root and tooth number (25). Description of root canal configuration is written as superscript after the tooth number on the course of the root canal starting from the orifices [O], passing through the canal [C], ending by the foramen [F], so it is single canal

Root canal morphology was recorded and classified according to the classification system proposed by Ahmed et al. in 2017. This classification system provides a standardized framework for describing root canal configurations, facilitating comparisons across studies. The obtained CBCT images were meticulously analyzed, with root canal morphology recorded for each maxillary premolar (Fig. 1). The images were divided into age groups (10 to 20, 21 to 30, 31 to 40, 41 to 50, 51 to 60, and 61 years above) and categorized by gender (males and females) to explore variations in root canal anatomy. Detailed analysis of each image was conducted to identify the number of roots, canals, and any anatomical variations present.

### Statistical analysis

Statistical analysis was performed using SPSS version 26 software. Descriptive statistics, including mean frequency and standard deviation, were calculated to summarize the data. The association between root canal morphology and age/gender was analyzed using the chi-square test or Fisher exact test, depending on the distribution of the data. Significance levels were set at *p* ≤ 0.05 to determine the statistical significance of the findings. Additionally, subgroup analyses were conducted to explore potential interactions between age, gender, and root canal morphology.

## Results

The distribution of maxillary premolars according to Ahmed’s classification was examined. Table 1 presents the distribution of premolars based on the classification categories. For right maxillary 1st premolars, the majority belonged to ^2^MPM^1^ B^1^ L^1^ (39.03%) and ^1^MPM^1^ (2.81%) categories. Similarly, for right maxillary 2nd premolars, ^2^MPM^1^ B^1^ L^1^ (39.08%) and ^1^MPM^1^ (17.85%) were the most prevalent categories.

**Table 1:** Distribution of maxillary premolars according to Ahmed’s classification

Classification	Right Max 1st PM		Right Max 2nd PM		Left Max 1st PM		Left Max 2nd PM	
	*n*	%	*n*	%	*n*	%	*n*	%
^1^ MPM ^1^	11	2.81	70	17.85	8	2.03	74	18.8
^2^ MPM ^1^ B ^1^ L ^1^	154	39.03	45	11.48	154	39.08	45	11.42
^3^ MPM MB ^1^ DB ^1^ L ^1^	1	0.25	3	0.77	2	0.5	2	0.5
^1^ MPM ^1–2−1–2−1–2^	1	0.25	1	0.25	0	0	0	0
^1^ MPM ^2−1−2−1^	0	0	1	0.25	1	0.25	0	0
^1^ MPM ^2−1^	4	1.02	2	0.51	6	1.52	2	0.5
^1^ MPM ^1–2−1^	22	5.61	52	13.26	26	6.6	52	13.2
^1^ MPM ^2−2^	11	2.81	9	2.3	9	2.28	5	1.26
^1^ MPM ^1–2^	3	0.77	1	0.25	4	1.01	3	0.76
^1^ MPM ^1–2−1–2^	1	0.25	1	0.25	0	0	0	0
^1^ MPM ^2−1−2^	0	0	0	0	0	0	1	0.25
Total	208	52.8	185	47.2	210	53.3	184	46.7

MPM- Maxillary Premolar

**Fig. 2:**
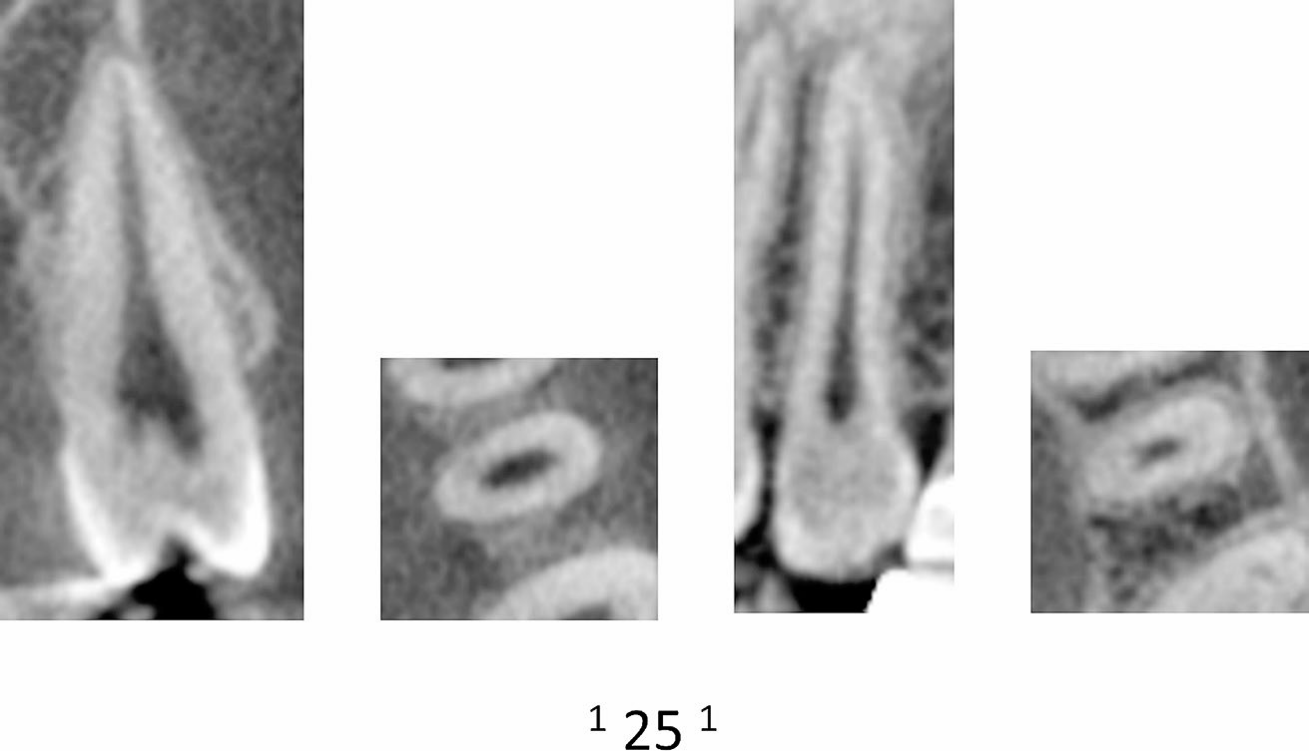
CBCT View (Sagittal and axial) of left maxillary second premolar showing the code ^1^MPM^1^

**Fig. 3:**
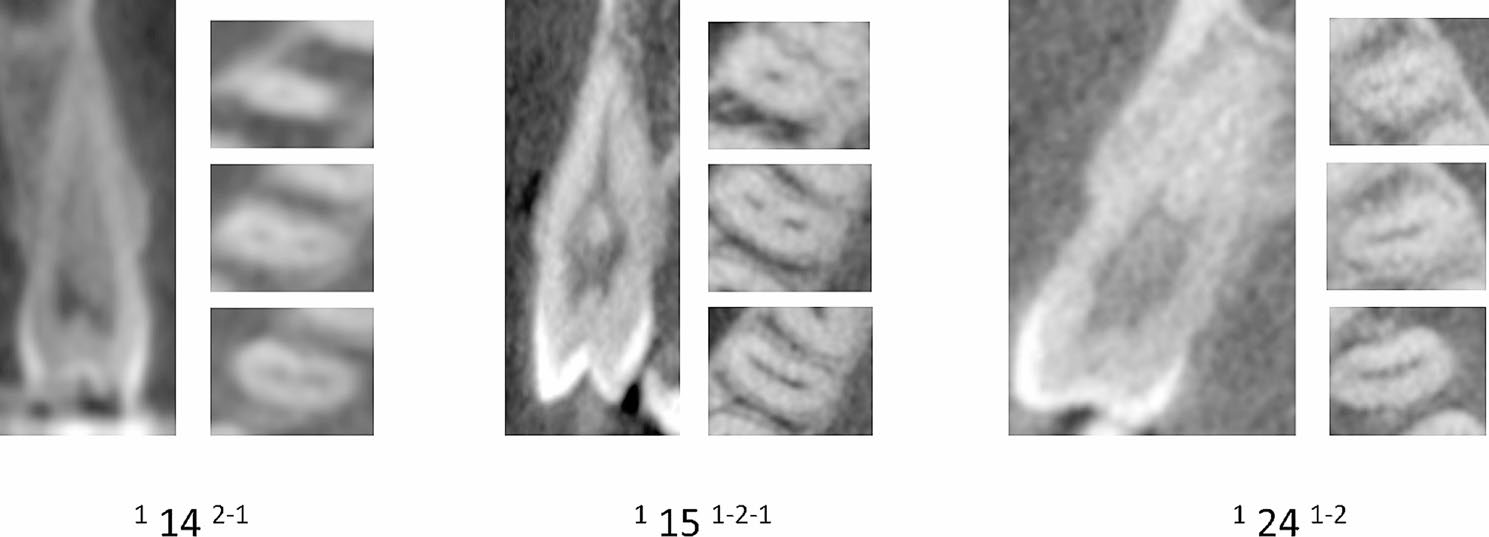
CBCT View (Sagittal and axial) maxillary first and second premolars showing the canal variations

**Fig. 4:**
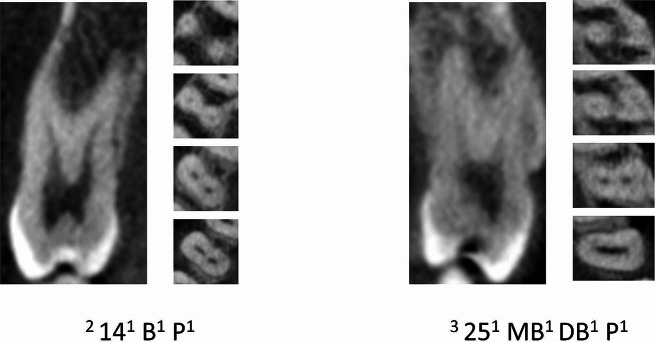
CBCT View (Sagittal and axial) maxillary first and second premolars showing the canal variations in more than one root

Table 2 displays the distribution of maxillary premolars based on the number of roots. The majority of premolars had two roots (73.33% for left maxillary 1st premolars, 24.45% for left maxillary 2nd premolars, 74.03% for right maxillary 1st premolars, and 24.32% for right maxillary 2nd premolars) (Figs. 2, 3 and 4).

**Table 2:** Distribution of maxillary premolars according to number of roots

Number of roots	Left Max 1^st^ PM	Left Max 2^nd^ PM	Right Max 1^st^ PM	Right Max 2^nd^ PM
One root	54	137	53	137
Two roots	154	45	154	45
Three roots	2	2	1	3
Total	210	184	208	185

Max PM- Maxillary premolar

Tables 3 and 4 present the distribution of left and right maxillary 1st and 2nd premolars, respectively, based on gender. In Table 3, significant gender differences were observed for the classification ^1^MPM^1^ (*p* = 0.515) and ^1^MPM^1–2−1^ (*p* = 0.010*) for both left maxillary 1st and 2nd premolars. The number of males and females for MPM^1^ in left maxillary 1st premolars was 121 and 88, respectively, while for ^1^MPM^1^ in left maxillary 2nd premolars, it was 111 and 72, respectively. Similarly, for ^1^MPM^1–2−1^ in left maxillary 1st premolars, the number of males and females was 3 and 3, respectively, whereas for left maxillary 2nd premolars, it was 30 and 21, respectively.

**Table 3:** Distribution of left maxillary 1^st^ and 2^nd^ premolar concerning gender

Classification	Left Max 1^st^ PM			Left Max 2^nd^ PM		
	Male	Female	*P*-value	Male	Female	*P*-value
^1^MPM^1^	6	2		36	38	
^2^MPM^1^ B^1^ L^1^	93	61		37	8	
^3^MPMMB^1^DB^1^ L^1^	1	1		1	1	
^1^MPM^2-1^	0	1		2	0	
^1^MPM^1-2-1^	3	3	0.515	30	21	0.010*
^1^MPM^2-2^	12	13		4	1	
^1^MPM^1-2^	3	6		1	2	
^1^MPM^2-1-2^	3	1		0	1	
Total	122	88		112	72	

*Significant value <0.05; Chi-square test, MPM and Max PM- Maxillary Premolar

**Table 4:** Distribution of right maxillary 1^st^ and 2^nd^ premolar concerning gender

Classification	Right Max 1^st^ PM			Right Max 2^nd^ PM		
	Male	Female	*P*-value	Male	Female	*P*-value
^1^MPM^1^	5	6		34	36	
^2^MPM^1^ B^1^ L^1^	100	54		38	8	
^3^MPMMB^1^DB^1^ L^1^	0	1		2	1	
^1^MPM^1-2-1-2-1-2^	1	0		0	1	
^1^MPM ^2-1-2-1^	0	0		0	1	
^1^MPM^2-1^	1	3	0.032*	2	0	0.003*
^1^MPM ^1-2-1^	10	12		33	18	
^1^MPM^2-2^	2	9		5	4	
^1^MPM^1-2^	2	1		1	0	
^1^MPM ^1-2-1-2^	1	0		0	1	
Total	122	86		115	70	

*Significant value <0.05; Chi-square test, MPM and Max PM- Maxillary Premolar

Table 4 indicates significant gender differences for the classification MPM^1^ (*p* = 0.032*) and ^1^MPM^1–2−1^ (*p* = 0.003*) in the right maxillary 1st premolars. The number of males and females for ^1^MPM^1^ in the right maxillary 1st premolars was 122 and 84, respectively, while for ^1^MPM^1^ in the right maxillary 2nd premolars, it was 115 and 70, respectively. Additionally, the number of males and females for ^1^MPM^1–2−1^ in right maxillary 1st premolars was 10 and 11, respectively, whereas, for right maxillary 2nd premolars, it was 33 and 18, respectively.

Tables 5 and 6 demonstrate the distribution of left and right maxillary 1st and 2nd premolars, respectively, based on age groups. In Table 5, significant differences were observed for the classification ^1^MPM^1^ (*p* = 0.053) and ^1^MPM^1–2−1^ (*p* = 0.002*) in left maxillary 1st premolars. The number of premolars in each age group for ^1^MPM^1^ in left maxillary 1st premolars ranged from 1 to 7, whereas for ^1^MPM^1–2−1,^ it ranged from 0 to 3. For left maxillary 2nd premolars, significant differences were observed for the classification ^1^MPM^1^ (*p* = 0.002*) and ^1^MPM^1–2−1^ (*p* = 0.002*). The number of premolars in each age group for ^1^MPM^1^ in left maxillary 2nd premolars ranged from 6 to 38, whereas for ^1^MPM^1–2−1,^ it ranged from 4 to 23.

**Table 5:** Distribution of left maxillary 1^st^ and 2^nd^ premolars concerning age

Classification	Left Max 1^st^ PM							Left Max 2^nd^ PM						
	AGE						*P*-value	AGE						*P*-value
	10-20	21-30	31-40	41-50	51-60	60+		10-20	21-30	31-40	41-50	51-60	60+	
^1^ MPM ^1^	1	3	3	0	1	0		13	23	16	9	7	5	
^2^ MPM ^1^ B ^1^ L ^1^	17	41	47	20	14	15		5	16	14	4	3	3	
^3^ MPM MB ^1^ DB ^1^ L ^1^	0	2	0	0	0	0		0	1	1	0	0	0	
^1^ MPM ^2-1^	0	1	0	0	0	0		0	0	1	1	0	0	
^1^ MPM ^1-2-1^	0	1	2	0	2	1	0.053	2	13	13	11	9	4	0.002*
^1^ MPM ^2-2^	3	7	5	5	3	3		0	3	1	0	1	1	
^1^ MPM ^1-2^	0	3	1	1	1	3		0	0	1	2	0	0	
^1^ MPM ^2-1-2^	0	2	1	0	1	0		0	0	1	0	0	0	
Total	21	60	59	26	22	22		20	56	48	27	20	13	

*Significant value <0.05; Chi-square test, MPM and Max PM- Maxillary Premolar

**Table 6:** Distribution of right maxillary 1^st^ and 2^nd^ premolars concerning age

Classification	Right Max 1^st^ PM							Right Max 2^nd^ PM						
	AGE						*P*-value	AGE						*P*-value
	10-20	21-30	31-40	41-50	51-60	60+		10-20	21-30	31-40	41-50	51-60	60+	
^1^ MPM ^1^	1	3	3	1	3	0		12	25	17	4	6	6	
^2^ MPM ^1^ B ^1^ L ^1^	17	39	45	22	13	18		7	13	14	5	3	3	
^3^ MPMMB ^1^ DB ^1^ L ^1^	0	1	0	0	0	0		0	2	1	0	0	0	
^1^ MPM ^1-2-1-2-1-2^	0	0	1	0	0	0		0	0	0	1	0	0	
^1^ MPM ^2-1-2-1^	0	0	0	0	0	0		0	1	0	0	0	0	
^1^ MPM ^2-1^	0	1	1	0	2	0	0.055	0	0	1	1	0	0	0.002*
^1^ MPM ^1-2-1^	3	7	8	1	0	3		1	15	12	7	7	10	
^1^ MPM ^2-2^	0	4	2	1	2	2		0	3	3	0	0	3	
^1^ MPM ^1-2^	0	2	0	0	1	0		0	0	0	1	0	0	
^1^ MPM ^1-2-1-2^	0	0	0	0	0	1		0	0	1	0	0	0	
Total	21	57	60	25	21	24		20	59	49	19	16	22	

*Significant value <0.05; Chi-square test, MPM and Max PM - Maxillary Premolar

In Table 6, significant differences were observed for the classification ^1^MPM^1^ (*p* = 0.055) and MPM^1^ (*p* = 0.002*) in the right maxillary 1st and 2nd premolars, respectively. The number of premolars in each age group for ^1^MPM^1^ in the right maxillary 1st premolars ranged from 1 to 6, whereas for ^1^MPM^1–2−1,^ it ranged from 0 to 15. For right maxillary 2nd premolars, significant differences were observed for the classification ^1^MPM^1^ (*p* = 0.002*) and ^1^MPM^1–2−1^ (*p* = 0.002*). The number of premolars in each age group for ^1^MPM^1^ in the right maxillary 2nd premolars ranged from 6 to 36, whereas for ^1^MPM^1–2−1^, it ranged from 3 to 15.

## Discussion

The present study aimed to investigate the root canal morphology of maxillary premolars using cone-beam computed tomography (CBCT) imaging. By analyzing a large sample size of CBCT images, we sought to provide a comprehensive understanding of the complex and variable root canal configuration in maxillary premolars, considering factors such as gender and age.

As mentioned in the literature [11, 19], our findings revealed a diverse range of root canal configurations in maxillary premolars. Multiple canals, isthmuses, and accessory canals in these teeth pose a challenge to endodontic treatment, as it necessitates thorough exploration, disinfection, and meticulous instrumentation [20]. Recognizing such complex anatomy underscores the importance of employing advanced imaging techniques, such as CBCT, to accurately visualize and assess root canal morphology [21, 22].

In our study, age emerged as a significant factor influencing the root canal morphology of maxillary premolars. The categorization into different age groups allowed for a nuanced exploration of these variations, corroborating previous research [23–25]. The age-specific analysis revealed noteworthy trends in the prevalence of certain root canal configurations. For instance, in left maxillary 1st premolars, the marginal significance (*p* = 0.053) for 1MPM1 suggests a potential shift in root canal anatomy with increasing age. This finding prompts further investigation into the underlying reasons for such variations across age groups. Similarly, the significant difference (*p* = 0.002*) observed in 1MPM1-2-1 in both left and right maxillary 1st premolars indicates distinct patterns in root canal morphology among different age brackets. This finding raises questions about whether these differences are attributed to developmental changes, wear and tear, or other factors associated with aging. These age-related changes can be attributed to factors such as dentin deposition and secondary dentin formation, which may alter the shape and complexity of the root canal system over time. Therefore, endodontists should consider these age-related variations when planning and performing root canal procedures, particularly in older patients [26]. Younger age groups may exhibit features associated with incomplete root development and open apices, while older age groups may show signs of maturation, closure of apices, and increased calcification [27]. The correlations between age-related changes in root canal morphology and systemic conditions enhance the clinical context. Systemic factors, such as hormonal changes, metabolic disorders, or medication use, may influence dental development and impact root canal anatomy differently across age groups [28]. Practitioners should consider these age-related nuances during treatment planning and execution, adjusting their approaches to accommodate the potential variations in root canal anatomy. For example, younger patients may exhibit different anatomical features compared to older individuals, influencing decisions related to instrumentation and obturation techniques.

Furthermore, our study identified gender-based differences in root canal morphology. This finding aligns with Ahmed et al. [19], who reported similar gender differences in maxillary premolars. Their study revealed a higher prevalence of multiple canals in males than females, which supports our observations of significant gender variations in root canal morphology. However, it is worth noting that Ahmed et al. did not mention the specific classification code ^1^MPM^1–2−1^ in their study, making a direct comparison somewhat limited.

Likewise, Cleghorn et al. [11] found that the prevalence of multiple canals in maxillary first premolars ranged from 30 to 73%, a range consistent with our findings. Shi et al., while studying the Chinese population [23], also noted significant differences in the number of roots and gender in both maxillary first and second premolars.

In a study conducted by Mashyakhy et al. [29] in a Saudi population, highly statistically significant differences in canal configurations were observed between genders in maxillary teeth. Similarly, Martins et al. [30] reported a gender difference in the root canal morphology of the Portuguese population. However, it is essential to mention that some contrasting results were found in specific subpopulations. For instance, no significant difference in root canal morphology was noted in the Malaysian subpopulation [31] and the German subpopulation [32].

In summary, our study adds to the existing body of literature by providing further evidence of gender-related variations in root canal morphology, and it is in line with previous research in this field.

This study’s utilization of CBCT imaging provided valuable insights into the three-dimensional morphology of maxillary premolars. CBCT has emerged as a powerful diagnostic tool in endodontics, enabling the visualization of intricate root canal anatomy [33]. Accurately assessing root canal morphology facilitates precise treatment planning, guiding clinicians in determining the appropriate access, instrumentation, and obturation techniques [34]. The present study has several advantages, reinforcing its conclusions’ reliability and veracity. First and foremost, a large sample size was used in the study, with 500 cone-beam computed tomography (CBCT) images in total, 1230 maxillary premolars included. This large sample size improves the study’s statistical power and broadens the applicability of the results to the intended population.

The study employed qualified endodontists and observers calibrated to evaluate root canal morphology to achieve precise and reliable analysis. To determine the classification of root canal morphology, 50 CBCT images were examined as part of the calibration process. The research boosted the consistency and accuracy of the results by creating a smooth decision-making process that reduced the possibility of observer bias.

In the present study, a standardized classification scheme was used. This classification system offers a reliable and standardized method for classifying root canal morphology. The study’s findings may be easily compared and integrated with those of other research utilizing the same approach because it used a recognized classification system. Understanding root canal morphology in maxillary premolars is ultimately enhanced by this, making it easier for future research and enabling meta-analyses.

Additionally, the study compared its findings to pertinent literature, enabling a thorough interpretation of the data in light of earlier research. The study offers important insights into the heterogeneity of root canal morphology in maxillary premolars by comparing the consistency or divergence of results across different populations and studies. The scientific knowledge base is expanded, and this topic is better understood thanks to the comparative method.

### Strengths of our study

One of the key strengths of our study is the large sample size, which enhances the statistical power and generalizability of our findings. Additionally, the utilization of cone-beam computed tomography (CBCT) imaging allowed for detailed three-dimensional analysis of root canal morphology, providing valuable insights into the complexity of maxillary premolars. Our rigorous calibration process, involving expert endodontists and observers, ensured the reliability and accuracy of our data collection and analysis. Furthermore, by considering age and gender variations, we were able to explore the influence of demographic factors on root canal anatomy, contributing to a more nuanced understanding of this topic.

### Limitations

Despite these strengths, our study also has several limitations that warrant consideration. Firstly, the retrospective nature of the study may introduce selection bias and limit the generalizability of the findings. Additionally, the study focused on a specific population, which may limit its applicability to other ethnic groups or regions. Furthermore, the reliance on CBCT imaging, while providing detailed anatomical information, is subject to radiation exposure and cost constraints. Moreover, the inclusion and exclusion criteria applied in the study may have inadvertently excluded certain teeth or patient populations, potentially affecting the representativeness of the sample.

Future research endeavors should explore the relationship between root canal morphology and treatment outcomes in maxillary premolars to enhance our knowledge further. Long-term follow-up studies can provide valuable insights into the success rates and potential complications associated with different root canal configurations. Furthermore, advancements in imaging modalities and treatment techniques, such as guided endodontics and regenerative approaches, hold promise for overcoming the challenges posed by complex root canal anatomy.

## Conclusion

This study provides novel insights into the root canal anatomy of maxillary premolars within the Saudi population, addressing a notable gap in the literature specific to this demographic. Through CBCT imaging and analysis of large sample sizes, the complex and diverse nature of root canal morphology in these teeth among Saudi individuals is elucidated. The findings underscore the importance of CBCT imaging in precise treatment planning and decision-making tailored to the Saudi population. Consideration of age and gender-related variations further enhances understanding and aids in personalized endodontic interventions within this demographic. Moving forward, these findings inform clinical practice within the Saudi community, emphasizing the need for customized approaches to optimize treatment outcomes.

## Data Availability

All data supporting the findings of this study are available from the corresponding author upon reasonable request.
